# Histone modifications in cocaine, methamphetamine and opioids

**DOI:** 10.1016/j.heliyon.2023.e16407

**Published:** 2023-05-19

**Authors:** Junzhe Cheng, Ziping He, Qianqian Chen, Jiang Lin, Yilin Peng, Jinlong Zhang, Xisheng Yan, Jie Yan, Shuliang Niu

**Affiliations:** a Department of Forensic Science, School of Basic Medical Science, Central South University, Changsha, Hunan, 410013, China; b Clinical Medicine Eight-Year Program, Xiangya School of Medicine, Central South University, Changsha, Hunan, China; c Department of Human Anatomy, School of Basic Medical Science, Xinjiang Medical University, Urumqi, 830001, China; d Department of Cardiovascular Medicine, Wuhan Third Hospital & Tongren Hospital of Wuhan University, Wuhan, Hubei Province, 430074, China

**Keywords:** Substance use disorder, Histone modifications, Cocaine, Methamphetamine, Opioids, Pharmacotherapy

## Abstract

Cocaine, methamphetamine and opioids are leading causes of drug abuse-related deaths worldwide. In recent decades, several studies revealed the connection between and epigenetics. Neural cells acquire epigenetic alterations that drive the onset and progress of the SUD by modifying the histone residues in brain reward circuitry. Histone modifications, especially acetylation and methylation, participate in the regulation of gene expression. These alterations, as well as other host and microenvironment factors, are associated with a serious of negative neurocognitive disfunctions in various patient populations. In this review, we highlight the evidence that substantially increase the field's ability to understand the molecular actions underlying SUD and summarize the potential approaches for SUD pharmacotherapy.

## 1 Introduction

Substance use disorder (SUD) is a neuropsychiatric disorder characterized by compulsive behavior of drug-seeking with adverse neurocognitive and behavioral consequences [1]. Repeated drug exposure elicits coordinated changes of gene expression in several brain regions, including nucleus accumbens (NAc), dorsal striatum (dStri), basolateral amygdala (BLA), ventral tegmental area (VTA), prefrontal cortex (PFC) and hippocampus (Hip) [[2], [3], [4]]. Although researchers have made breakthroughs in recent years, the molecular mechanisms underlying SUD at the transcriptional level remain unclear.

Epigenetics describes the heritable reversible alterations in gene expression without changing the nucleotide sequences [5]. Epigenetics primarily encompasses histone modifications, DNA methylation and non-coding RNAs [6]. In recent decades, increasing evidence has indicated the essential role of epigenetics in SUD [7]. Drug abuse induces life-long cerebral reward system abnormality by epigenetic modifications in neural cells [8]. Especially, the role of histone modifications is extensively elaborated in SUD [9]. The involvement of histone modifications has been demonstrated in multiple aspects of neuronal function and synaptic plasticity during drug exposure. Herein, we present recent advances on the neurobiological implications of histone modifications for cocaine, methamphetamine and opioid abuse. We highlight areas where future research will extend this fundamental knowledge of SUD and exploit it for emerging applications in pharmacotherapy.

## 2 Histone modifications

Histone modifications make a difference in gene expression involved in SUD and regulate addictive behavior by remodeling the basic unit of chromatin, nucleosome. In eukaryotes, nucleosome core particles (NCP) are formed by the long genomic DNA wrapped around an octamer of two distinct copies of four types of histones (H2A, H2B, H3, H4) [10]. Histones are a family of small basic proteins with a globular domain and histone tails, which contain a flexible charged –NH_2_ terminus [11].

Histone modifications are covalent modifications where the N-terminal tails of amino residues in histone protein undergo modifications by addition or deletion of different functional groups, such as acetylation, methylation, ubiquitination, phosphorylation, SUMOylation, ADP ribosylation, deamination and proline isomerization [12]. These modifications change the nucleosome charge following disrupting electrostatic interactions between the modified residues and nucleic acids [13]. Histone modifications change the 3-dimensional architecture of chromatin and integrate the abundant genetic and environmental message into phenotype [14]. The alteration of chromatin structure is carried out by opposing editing processes of enzymes called ‘writers’ and ‘erasers’ [15]. Writers add various functional groups to histone amino residues of promoters and enhancers and attenuate the binding between DNA and histone to facilitate transcriptional activation, such as histone acetyltransferase (HATs). Erasers remove the modification of writers and govern transcriptional repressions, such as histone deacetylases (HDACs) [16]. Histone modifications upgrade the accessibility of plasticity-related genes and influence the activity of critical transcription factors through interaction with writers and erasers [17]. Then it recruits histone-modification-recognizing proteins called “readers” and transcriptional co-activators conversely. The genes required for consolidation transform to a permissive transcriptional state by histone modification [18]. The modifications of proteins are a major source of transcription diversity. This aberrant regulation is a negative outcome of drug abuse, damaging normal gene expressions and cellular functional operations. Aberrant deciphering functions of histone could induce drug-elicited neurotoxicity caused by histone modification.

Histone acetylation is a dynamic reversible mechanism with the transition from one chromatin architecture to a more open state, and it could influence gene expression by changing chromatin structure to make transcriptional factors accessible or inaccessible to genes [19]. Histone acetylation is regulated by the opposing activities of HATs and HDACs [19]. HATs and HDACs are usually found in association with other proteins in complexes. HATs catalyze the addition of acetyl groups from acetyl-CoA to the ε-amino group of lysine side chains on histone terminals generally, particularly histone H3 and H4 [20], leading to neutralizing the positive charge on the lysine residue, attenuating the interactions between histone and DNA double helix, and making highly condensed chromatin structure slack. So DNA could potentially have more access to transcriptional factors and RNA polymerase II for transcription [21]. HATs have three prominent families: GNAT (HAT1, GCN5, PCAF), MYST (Tip60, MOF, MOZ, MORF, HBO1) and p300/CBP. In comparison, HDACs have the opposite effect: removing the acetyl groups and condensing the chromatin [22]. HDACs could be divided into four major families, including Class I (HDACs 1, 2, 3, 8), Class IIA (HDACs 4, 5, 7, 9), Class IIB (HDAC 6, 10), Class III or sirtuins (SIRT1-7) and Class IV (HDAC11) [23]. Class I, II and IV HDACs are Zn^2+^-dependent enzymes and only expressed in the brain, primarily by neurons, while sirtuins are NAD^+^-dependent enzymes [24]. These two antagonistic enzymes jointly control histone acetylation processes.

Histone methylation is another distinctive histone modification, which reversibly changes the chromatin structure by adding or removing methyl groups on amino acid residues [25]. The category of modified residues and the number of methyl groups determined activation or inhibition of gene expression. Basic residues, such as lysine, arginine and histidine, can be methylated by 1–3 methyl groups in histone proteins. In general, the addition of methyl groups is associated with transcription repression. The methylations in H1, H2A, H2B, H3 and H4 have been observed, but most extensive research concentrate on histone methylation sites in H3 and H4. Histone methyltransferases (KMTs) and lysine demethylases (KDMs) jointly regulate the methylation and demethylation of residues on histone to control the cell cycle, transcription and neuronal plasticity [26].

## 3 Cocaine and histone modifications

According to the United Nations Office on Drug and Crime, roughly 20 million people worldwide had used cocaine in 2019, and the number of people who used cocaine increased by 22% between 2010 and 2019 [27]. Patients who abuse cocaine always suffer cognitive, behavioral, and physical disorders due to neural cell damage and metabolic dysfunction in the central nervous system (CNS) [28]. Cocaine use induces alterations in human brain regions and changes neuronal plasticity and drug-related memory reconsolidation [29,30]. Cocaine experience changes the synaptic transmission by regulating neurotransmitter transporters levels, producing persistent neural adaptation and synaptic adaptation underlying reward circuitry, and meanwhile contributing to the development and maintenance of cocaine use disorders (CUD) [31]. The normal functions of transporters on the presynaptic membrane, including dopamine reuptake transporters (DAT) [32], noradrenaline reuptake transporters (NET) [33], and serotonin reuptake transporters (SERT) [34], are interfered by cocaine with a series of adverse cognitive and physical responses. Some of these changes are elicited by epigenetic alterations, including histone modifications [35].

### 3.1 Histone acetylation and methylation in brain regions during cocaine exposure

Cocaine leads to transcription-dependent neuroplasticity in brain reward circuitry. Histone acetylation in cocaine exposure mainly occurs on H3 and H4. The H3K9, H3K14, H4K5, H4K8, H4K12 and H4K16 are the most widely studied acetylation sites [36]. Previous studies have indicated the essential role of brain nuclei heterogeneity in many regions of the reward pathway, and the variety of histone modifications at the promoters of specific genes interrelates with the diversity of the cerebrum closely (e.g., VTA, mPFC, NAc).

In NAc, repeated cocaine exposure causes increases of H3Ac/H4Ac at 1651 gene promoters and decreases at 206 gene promoters due to H3/H4 hypoacetylation [37]. Many epigenetic enzymes are involved in these processes. For example, histone acetylation in NAc is associated with CREB-binding protein (CBP), a histone acetyltransferase (HAT) [38], which mediates transcriptional activation and might play a vital role in long-term memory formation. CBP generally binds with phosphorylated CREB to regulate targeted gene activation. The deletion of CBP attenuates the increases of H3K14Ac and H2BK12Ac in NAc caused by acute and chronic cocaine administration. Further, a bilateral deletion of CBP in the NAc attenuated the rewarding effects of cocaine [39].

In VTA and mPFC, the enrichment of CBP at the brain-derived neurotrophic factor (Bdnf) promoter occurs in cocaine withdrawal, and promotes Bdnf transcription with the increased level of H3K9/14Ac at the Bdnf promoter [40]. Bdnf is a family of secreted proteins as a signal for proper axonal growth, which plays an important role in the development and maintenance of dopaminergic, GABAergic and serotonergic neurons [41]. Acute and chronic cocaine exposure induced increases in Bdnf levels in both the development and extinction stages of CUD, and Bdnf increases enhanced cocaine craving and stress-induced reinstatement after withdrawal [42].

Other modified sites, including H3K4Me1 and H3K4Me3, increase the global level of gene transcription, whereas enrichment of H3K9Me3 and H3K27Me3 at specific gene regions represses transcription [43]. Besides lysine (K) methylation, histone arginine (R) could be methylated by protein-R-methyltransferase (PRMT1 and 6) and generate H3R2Me2 and H4R3Me2 in NAc after repeated cocaine exposure [44,45].

Overall, the substantial numbers of modification sites and differential epigenetic enzymes in histones illustrate the potential complexity in the context of the cocaine reward circuitry. Further, the manipulation of epigenetic enzymes indicates a novel frontier for medication development. Recent studies have begun to search for effective epigenetic inhibitors to treat the behavioral disorder of cocaine addicts.

### 3.2 Epigenetic pharmacological treatment of CUD

The regulators of histone acetylation, HATs and HDACs, function as ‘writers’ and ‘erasers’ in maintaining brain homeostasis in various neurodegenerative diseases and abuse of drugs [46]. The primary goals of pharmacotherapy include alleviating drug craving and intake, abating withdrawal symptoms, and avoiding relapse for the patient. The role of HDACs has been extensively studied in CUD preclinical treatment [47,48]. HDAC inhibitors have been shown to be critical regulators in a manner resistant to relapse that contributes to the extinction of cocaine-seeking behavior. HDAC inhibition may disrupt memory reconsolidation or performance, resulting in the absence of drug-seeking behaviors [49]. For example, the HDAC3-selective inhibitor, RGFP-966, attenuates cocaine-related responses in enhancing long-term memory processes, including facilitating the extinction of cocaine-seeking and relapse-like behaviors [50]. Hence, pharmacologic manipulation of these regulators could help fill a void aimed at facilitating cognitive-behavior treatments to reduce cocaine abuse and relapse in drug abusers.

With the prominent progress in histone acetylation, the role of the bromodomain and extra terminal (BET) family has been intensively investigated in the epigenetic treatment of cocaine and other psychostimulants [51]. BET protein binds acetyl-lysine modifications on histone residues (e.g., H3K27Ac, H4K5Ac, H4K12Ac) as epigenetic ‘reader’ proteins, bromodomain‐containing protein (BRD2, BRD3, BRD4 and BRDT). BET family constructs a scaffold for recruiting additional protein complexes to regulate the chromatin dynamics and transcriptional activity. During cocaine exposure, the level of BET protein increased and it recruited to the Bdnf promoter in NAc [52]. The administration of BET inhibitors attenuates the transcriptional activity and behavioral response to cocaine exposure [53]. JQ1, a wide-studied pan-BET inhibitor, has been shown to be effective in various cocaine-induced symptoms, affecting contextual recognition and learning slightly. However, chronic JQ1 use may impair the novel object recognition memory. JQ1 could also block long-term memory formation during the process of memory consolidation. Pan-BET inhibitors may have undesirable effects on learning and memory and potential toxic liabilities in patients [54]. Pan-BET inhibitors may have undesirable effects on learning and memory and potential toxic liabilities in patients. With the development of further research on histone acetylation, the role of selective BET inhibitors has been recognized in epigenetic pharmacotherapy. In comparison, RVX-208, a novel BRD2-selective BET inhibitor, only modestly regulated BET-dependent gene expression and induced unique transcriptional activities [55]. RVX-208 could dose-dependently decrease multiple gene expressions in NAc and attenuate the responses without affecting behaviors like elevated zero mazes, locomotor activity, or novel object recognition memory [56].

Till now, none of the medications has yet been approved in clinical therapeutics for CUD [57]. Given the heterogeneity of CUD in human patients, the results of systemically administered epigenetic enzyme inhibitors may have interfered with clinical trials, and the epigenetic pharmacotherapy for CUD is still limited. Identifying the subgroups of different cocaine abusers and designing medications for targeted subgroups are rational routes for CUD treatment. Many pre-clinical studies have applied new cell and gene type-specific techniques to identify long-acting and effective medications. These findings provide new potential therapeutic strategies for cocaine pharmacotherapy and appear to be a safe and effective clinical treatment approach.

### 3.3 New histone modification forms in SUD

Besides acetylation and methylation, some new classes of histone modification have been gradually identified in SUD, including cocaine and opioids. A drug generally regulates epigenetic modifications and transcriptional activities in two ways: as a direct approach of the drug activating or repressing its specific molecular targets and related downstream signaling cascades, or an indirect effect in neurotransmitters signaling and the downstream cascades. Drug abuse could make inferences on common neurotransmitters in brain regions, including dopamine, noradrenaline and serotonin [58]. Recently, neurotransmitters have been proven to participate in histone modification as functional groups. Inconsistent with previous forms of histone modification, the enrichment of neurotransmitters has been observed in glutamate (Q) rather than lysine primarily. The first observed modified site is histone H3 glutamine 5 dopaminylation (H3Q5Dop), which plays a critical role in regulating gene expression and transcriptional plasticity [59]. Lepack et al. [59] repressed the accumulation of H3Q5Dop in VTA during cocaine withdrawal (30 days). As a result, dopamine release was downregulated in NAc and motivation to self-administered cocaine was decreased, which indicated that dopaminylation contributes to the carving and seeking behaviors of cocaine abuse. A possible explanation was that dopaminylation inhibition elicited a “homeostatic brake” and decreased NAc Src signaling in NAc D2-MSNs, which subsequently attenuated cocaine-seeking behaviors [60]. Apart from CUD, histone dopaminylation has also been observed in the heroin withdrawal process in VTA [61]. Like dopaminylation, serotonylation also occurs on histone H3 glutamine 5 (H3Q5Ser) [62]. H3K4Me3-marked histone could be subsequently serotonylated by transglutaminase 2 (TGM2) and generated a combinatorial H3K4Me3Q5Ser, which played a crucial role in neurotransmission and cellular signaling [63]. These novel forms of histone modification reveal the crucial role of neurotransmitters in relapse-related histone modifications, and establish a neurotransmission-independent interrelation with transcriptional activities and synaptic plasticity related to SUD, which possibly provide a new research direction to the molecular actions of SUD.

## 4 Methamphetamine (METH) and histone modifications

Methamphetamine, which belongs to amphetamine-type stimulants (ATS), is a widely abused pharmacologic psychostimulant with complicated neurotoxicity in the central nervous system (CNS) [64]. This compound rapidly passes through the blood-brain barrier and persists within the CNS readily due to its high lipid solubility, eliciting severe neurotoxicity, oxidative stress and necroptosis [65]. As said by the United Nations Office on Drug and Crime, 27 million people abuse ATS throughout the globe with a significant annual growth rate [27]. And long-term abuse of METH could disrupt the bio-pathological neuroadaptations and result in METH-use disorders (MUD) with complex cerebrovascular and cardiovascular symptoms [64]. MUD is accompanied by multiple alterations in gene transcriptions and protein expression related to epigenetic mechanisms within specific brain regions. The present evidence indicates that histone modifications play a significant role in the transition from recreational methamphetamine use to MUD in humans, particularly histone acetylation and methylation.

### 4.1 Histone acetylation and methylation in brain regions during METH exposure

Similar to cocaine, METH abuse elicits time-dependent changes in the acetylation of H3 and H4 levels associated with the expression of various enzymes in brain regions [66]. Previous studies have investigated the trend of acetylation levels in reward circuitry, but the experimental results show high complexity and diversity in specific endpoints of different brain regions. In NAc, the exposure of METH could induce differential acetylation changes on various histone lysine residues by regulating the protein levels of histone deacetylases [67]. Acute METH-exposure resulted in decreases in H3Ac (H3K9Ac, H3K18Ac) whereas increases of H4Ac (H4K5Ac H4K8Ac H4K16Ac) [68]. In PFC, METH induced significant hyperacetylation of 275 genes, including 821 H3 hyperacetylated genes and 10 H4 hyperacetylated genes [69]. In dStri, acute and chronic METH exposure caused a global increase in H4K5Ac at the transcriptional start sites [70]. By contrast, chronic METH caused a decrease in the level of histone H4Ac (H4K5Ac, H4K12Ac, H4K16Ac) at glutamate (GLUT) receptors promoters, which attenuated the expression of alpha-amino-3-hydroxy-5-methyl-4-isoxazole propionic acid (AMPA) receptor and N-methyl-d-aspartate (NMDA) receptor and elicited a series of abnormities in neural cell (e.g., oxidation, excitotoxicity, and neuroinflammation) [71,72]. In striatum, H4 hypoacetylation might be a primary determinant in METH-induced decreased expression of GLUT [73]. The decrease of acetylation levels at these sites represses a relaxation of chromatin structure, leading to a decrease of mRNA and protein levels of AMPAr and NMDAr. Consequently, these changes elicit maladaptive plasticity within striatal, limbic and isocortical brain areas, and impair normal intense emotional and memory retrieval functions [66]. Animal models of MUD have indicated that these changes might induce increasing METH-craving behaviors and relapse in human addicts, and the formation of MUD might be secondary to complex histone modifications and transcriptional changes in brain reward circuitry.

However, many different and even reverse results could be observed in animal model experiments of METH. For instance, González et al. [74] reported that a single METH injection increased levels of total H3 acetylation and decreased H4 acetylation. However, in another paper, González et al. [75] reported that in the medial prefrontal cortex, both H3Ac and H4Ac showed inconsistent changes in specific protein levels after repeated METH use. Repeated METH injection decreased the H3Ac enrichment at the promoters of DA receptor (Drd2), orexin receptors (Hcrtr1 and Hcrtr2), histaminergic receptor (Hrh1) and glutamatergic receptor (NMDA Grin1), and increased H4Ac enrichment at promoters of DA receptor Drd1, histamine receptor Hrh1 and NMDA receptor subunit Grin1 [75]. The diversity of H3 and H4 acetylation between single and repeated exposure may be responsible for the increasing dose craving in drug-seeking behavior of MUD. And the histone-lysine N-methyltransferase 2A (KMT2A), which is associated with memory formation and maintenance [76], has been increased in the METH CPP animals with the level of H3K4Me3 increasing in the NAc [25]. However, in the dStri, H3K4Me3 is not observed on the promoters of immediate early genes in METH self-administration models [77]. These different and even adverse results indicate that multiple factors might induce diverse histone modifications and transcriptional changes in animal models, such as the different species of animal models, the different roles of brain regions in responses to the substances and the different administration patterns. And these regulations in transcription level are responsible, in part, for influencing the dynamic plasticity and range of behavior and physiology during acute and chronic METH administration.

### 4.2 Effects of METH on epigenetic regulators

The changes in HDAC level have high heterogeneity in brain subregions and SUD periods during METH use. In NAc, HDACs expression was decreased globally after acute and chronic METH exposure [78]. But Nestler reported that a single injection with a large dose of METH increased HDAC2 expression in NAc of rats [79]. In PFC, METH increased HDAC1 and HDAC2 levels globally [69], whereas González et al. [74] reported that single-dose METH increased H4Ac at HDAC1 and decreased it at HDAC2. In DS, class I HDACs (HDAC1, HDAC2) mRNA levels displayed a significant decrease [10]. Further, HDAC knockout animal models indicate the role of HDACs in the effects of METH. For example, the knockout of HDAC5 in DS significantly decreased the METH-craving behavior, and these alterations increased the level of HDAC1 during METH withdrawal [80]. The exposure dose and time of METH for animal models have diverse influences on HDAC response in various histone acetylation sites. It may be challenging to search possible targets in METH-elicited alterations in the future development of pharmacotherapies. Indeed, traditional, single neurotransmitter receptor-targeted medication may be difficult to reverse the intricate pathological neuronal connectivity and synaptic plasticity. However, compared to cocaine, the epigenetic-based therapies for MUD are still limited due to the complexity of MUD. The mechanism and concrete function of HDACs in METH action are still unclear. More research on the roles of HDACs in METH use need to be conducted before discovery of epigenetic drugs in treating MUD.

## 5 Opioids and histone modifications

Overdose deaths caused by opioid involvement in drug abuse increased from 34.5% in 2010 to 53.5% in 2019 [81,82]. Abuse of opioids, such as morphine and heroin, produces several alterations in brain reward-processing networks. This effect is elicited by divergent mechanisms of epigenetic changes distributed across different brain regions and nerve cell types [83]. The μ-opioid receptors (MORs) on neuron or non-neuronal cells within the VTA mediate various signaling cascades and neuronal responses by opioid exposure [84]. Consequently, repeated opioid induces a series of physical and psychological symptoms, including memory dysfunctions, immunity decline and cardiovascular disease [85,86]. Several epigenetic modifications have been observed and linked to the abnormalities in specific gene expression in opioid exposure and withdrawal.

The MORs on the neuronal cells of NAc could identify and bind opioid, and subsequently elicit distinct neuronal epigenetic regulations throughout other brain regions. Repeated opioid exposure, particularly morphine and heroine, induces hyperacetylation at H3K9, H3K14, H3K18 and H3K27 in SA models [87]. It is reported that glutamatergic transcriptional changes are associated with heroin-related histone H3 hyperacetylation underlying SUD behavior, which increases the chromatin accessibility at glutamatergic genes in NAc [88]. Egervari et al. reported that heroin exposure consistently increases H3K27Ac levels in striatum of human heroin users and SA rats, and the level of H3K27Ac is positively correlated with the years of human heroin users [88]. In addition to H3, the hyperacetylation at H4 (H4K5 and H4K8) in NAc could increase the heroin-seeking behavior of rats [89]. Further, chromatin accessibility mapping with ATAC-seq confirmed that heroin-induces histone hyperacetylation promotes an increasingly accessible chromatin state, which ultimately facilitates higher levels of neuroplasticity-related gene transcriptional activity [88].

Compared to acetylation, the understanding of how opioids regulate histone methylation is superficial. Only specific histone tail residues---H3K9 and H3K27 have been identified in opioid treatment [[90], [91], [92]]. Repeated opioid exposure reduces H3K9Me2 in NAc and the central nucleus of the amygdala, and chronic opioid treatment appears to promote transcriptional activities [92]. Polycomb repressive complex 2 (PRC2) participates in H3K27 trimethylation, inducing the chromatin in transcription silent at developmental genes. During morphine treatment, PRC2 complex is targeted to selected promoters, and causes a global reduction in H3K27Me3 levels. Following morphine withdrawal, the levels of enhanced promoter H3K27Me3 return to normal first in general level. It indicates that H3K27Me3 is involved in the formation of morphine-induced characteristic changes, but not in their maintenance [90].

Epigenetic mechanisms regulate several neural circuits and molecular pathways, and provide a novel approach for improving cognitive dysfunction elicited by opioids. Dynamical regulation of chromatin structure by HAT and HDAC induces multiple transcriptional activities and related synaptic plasticity. Manipulating the level of epigenetic regulators can also change the opioid-related behaviors. The administration of HDAC inhibitors enhances long-term memory and locomotor sensitivity in abuse of opioids. The bromodomain inhibitor JQ1 (2 μl of 20 μmol/l) was injected before heroin SA, and it interrupted the read-out of functional group and decreased the heroin-seeking behaviors [88]. However, the systemic and intra-NAc administration of JQ1 did not affect the acquisition of morphine CPP in mice, while it also attenuated cocaine-seeking behaviors [93]. These different results reveal a complex interrelation between the regulators of epigenetic modification and carving behaviors of opioids, which indicate the significance of identifying the distinction between morphine and heroin in the potential treatment of opioids use disorders.

## 6 Conclusion and future perspectives

**Fig. 1:**
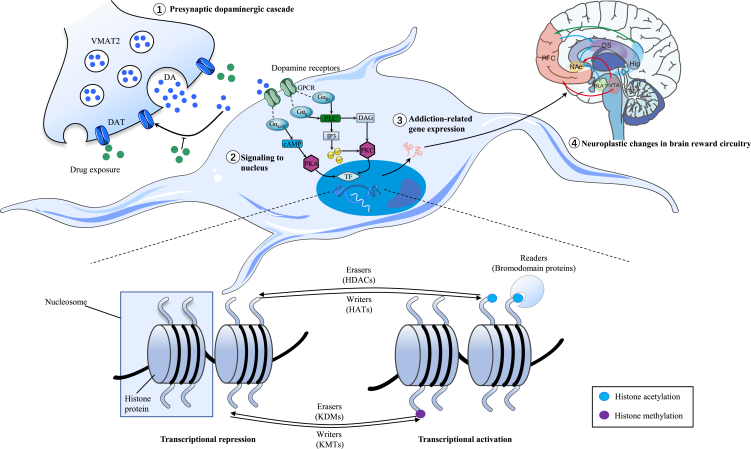
Psychostimulants elicit transcription-dependent neuroplasticity in brain reward circuitry: ① Drugs cause synaptic plasticity by regulating presynaptic and postsynaptic dopaminergic signaling. ② Transcription activities in neural cells are changed through a series of cascades to the nucleus. ③ Histone modifications regulate the accessibility of plasticity-related genes and influence the activity of critical transcription factors through interaction with epigenetic regulators called writers and erasers. Then it recruits reader proteins and transcriptional co-activators conversely and promotes gene expression. ④ These changes regulate neuroplasticity in reward circuitry, such as NAc, VTA, mFPC, etc. AbbreviationsBLA: basolateral amygdala; cAMP: cyclic adenosine monophosphate; DA: dopamine; DAG: diacylglycerol; DAT: dopamine transporters; DS: dorsal striatum; GPCR: G protein-coupled receptor; HATs: histone acetyltransferases; HDACs: histone deacetylases; Hip: hippocampus.IP3: inositol triphosphate; KDMs: lysine demethylases; KMTs: histone methyltransferases; NAc: nucleus accumbens; PFC: prefrontal cortex; PKA: protein kinase A; PKC: protein kinase C; TF: transcriptional factor; VMAT2: vesicular monoamine transporter 2; VTA: ventral tegmental area.

In general, acute and repeated exposure to METH, cocaine and opioids, increases the level of permissive histone acetylation, and decreases the level of repressive histone methylation in the reward system, including NAc, VTA, PFC and other brain regions (Table 1). Current evidence suggests several properties of histone modifications in SUD: 1) drug abuse generally alters the levels of histone modifications in the reward circuitry, thereby activating or repressing the expression of neuroplasticity-related genes, including IEGs and others related to dopaminergic and glutamatergic paths (Fig. 1); 2) the ‘writers’ and ‘erasers’ of histone modifications are closely associated with the behavioral response to SUD. Manipulating the activities of these enzymes could regulate underlying drug-related changes in synaptic plasticity in the brain reward regions; 3) the functional interplay between the alterations of histone and drug abuse is not consistent in any condition. The consequences of these modifications in specific genes are associated with such factors as psychotropic drugs, animal species, experimental methods, duration of exposure, brain regions, neural cell types and specific modified sites. Therefore, although we have understood the global regulation in several brain areas, we cannot assume that specific epigenetic or transcriptional changes will generalize from one drug class to another. It is still challenging to conclude how addictive drugs reprogram the epigenetic and transcriptional landscape in the reward circuitry at this stage.

**Table 1:** Histone modifications regulated by substance use disorders.

Epigenetic mark	Brain region	Drug	Change	Model	Target gene	Reference
Pan-H3ac	Striatum	Heroin	↑	Human addicts, Rats IVSA		[88]
	NAc	METH	↑	Mouse, repeated + CPP	*Nrxn, Syp, Dlg4, Gria1, Grin2a, Grin2b, Camk2a, Creb, Cdk5*	[100]
	NAc	Cocaine	↑	Rats & Mouse, repeated + CPP	*cFOS, Cbp, Bdnf, FosB, Cdk5, CaMKlla, Gria2, Dlg4*	[39,[101], [102], [103]]
			No Δ	Mouse, repeated + CPP	*cFos*	[39]
	VTA	Cocaine	↑	Rats, repeated + WD	*Bdnf*	[40]
Pan-H3phosphoac	NAc (not PFC)	Heroin	↑	Mouse, repeated + CPP		[104]
H3K9Ac	NAc	METH	↓	Rats, acute		[105]
		Cocaine	↑	Mouse, repeated + CPP	*Ehmt2, Suv39h1*	[106]
	LC, VLO	Morphine	↑	Rats, repeated	*Bdnf*	[107,108]
H3K14Ac	NAc	Cocaine	↑	Mouse, repeated + CPP	*Ehmt2, Suv39h1*	[39,106]
				Mouse, acute		[39]
	dStri	METH	↑	Mouse, repeated		[109]
	BLA	Morphine	↑	Mouse, repeated + CPP, WD	*Bdnf, FosB, Creb*	[110]
H3K18Ac	NAc	METH	↓	Rats, acute		[105]
		Heroin	↑	Rats, repeated + WD		[89]
H3K23Ac	NAc/dStri	Heroin	↑	Human addicts (not correlated with use history)		[88]
H3K27Ac	NAc/dStri114	Heroin	↑	Human addicts, Rat IVSA	*Glutamate signaling, GRIA1*	[88]
	NAc	Cocaine	↑	Mouse, repeated + WD	*Nr4a1, Carpt*	[111]
H4K5Ac	NAc	METH	↑	Rats, acute		[68]
		Heroin	↓	Rats, repeated + WD		[89]
	dStri	METH	↑	Rats, repeated	*GluA1, GluA2 and GluN1*	[73]
H4K8Ac	NAc	METH	↑	Rats, acute		[68]
		Cocaine	No Δ	Mouse, repeated + CPP	*cFos, Nr4a2*	[112]
		Heroin	↑	Rats, repeated + WD		[89]
H4K12Ac	NAc	Cocaine	↓	Mouse, repeated + CPP		[39]
	dStri	METH	↓	Rats, repeated	*GluA1, GluA2 and GluN1*	[73]
			↑	Mouse, acute & repeated		[109]
H4K16Ac	dStri	METH	↓	Rats, repeated	*GluA1, GluA2 and GluN1*	[73]
	NAc	Cocaine	↑	Mouse, acute & repeated + CPP		[113]
H3K4Me2	NAc	METH	↑	Mouse, repeated + WD		[114]
H3K4Me3	NAc	METH	↑	Mouse, repeated + WD	*CCR2*	[114]
H3K9Me1	NAc	Morphine	No Δ	Mouse, repeated		[91]
H3K9Me2	NAc	Cocaine	↓	Mouse, repeated + CPP		[39,115]
	dStri	Morphine	↓	Mouse, repeated	*FosB, Bdnf, glutamate signaling genes*	[91]
		Morphine	↓	Mouse, repeated		[92]
H3K9Me3	NAc	Cocaine	↑	Mouse, repeated & acute		[116]
			↓	Mouse, repeated or acute + WD		[116]
		Morphine	No Δ	Mouse, repeated	*Bdnf*	[91]
	VTA, LC	Morphine	↓	Mouse, repeated + WD	*Bdnf*	[107]
H3K27Me2	NAc	METH	↓	Mouse, repeated + WD		[117]
H3K27Me3	NAc	Morphine	No Δ	Mouse, repeated		[91]
	NAc	METH	↑	Rats, repeated	*Crh, Avp*	[118]
H3Q5Dop	VTA	Cocaine	↑	Rats, repeated		[59]
		Heroin	↑	Rats, repeated		[61]

Abbreviations.

2Me: demethylation.

3Me: trimethylation.

Ac: acetylation.

AMY: amygdala.

Arrows (↑↓): an increase or decrease.

Bdnf: brain-derived neurotrophic factor.

CaMKII: calcium/calmodulin-dependent kinase II alpha.

Cbp: CREB-binding protein.

CCR2: C–C chemokine.

cFos: FBJ murine osteosarcoma viral oncogene homolog.

CPP: conditioned place preference.

Dlg4: discs, large homolog 4.

dStri: dorsal striatum.

Ehmt2: euchromatic histone-lysine N-methyltransferase 2.

Gria1: glutamate receptor, ionotropic, AMPA 1.

Gria2: glutamate receptor, ionotropic, AMPA 2.

Grin1: glutamate receptor, ionotropic, N-methyl d-aspartate 1.

Grin2b: glutamate receptor, ionotropic, N-methyl d-aspartate 2B

IVSA: intravenous self-administration.

LC: locus coeruleus.

METH: methamphetamine.

mPFC: medial prefrontal cortex.

NAc: nucleus accumbens.

No △: no effect in specific modifications.

Nr4a2: nuclear receptor subfamily 4, group A, member 2.

Suv39h1: suppressor of variegation 3–9 homolog 1.

VLO: ventrolateral orbital cortex.

VTA: ventral tegmental area.

WD: withdrawal.

Epigenetic therapeutics have appeared to be promising candidates for reliving the symptoms of SUD. Histone modifications may regulate the gene expression by the ‘writers’ and ‘erasers’. Therefore, these enzymes could be potential targets for SUD pharmacotherapy (Table 2). Promising effects have been observed in decreasing the addictive behaviors of several classes of drugs [94]. Despite important advances in epigenetic therapeutics, several challenges are unsolved nowadays. For example, current epigenetic inhibitors are pan-BET inhibitors and not selective for specific molecule, but most studies of BET therapeutics concentrated on BRD4 in histone modification. Other BET proteins like BRD2 and BRD3 are ignored, and they may also play an important role in CNS-related diseases [51]. Hence, more studies are needed to elucidate the mechanisms of upstream signals in histone modifications to individual BET proteins. Further, some adverse side effects are observed in SUD therapeutic processes, such as cognitive impairment and memory decline. The use of epigenetic inhibitor in a therapeutic context is still challenging due to the relatively limited availability of epigenetic inhibitor isoform and a high risk for adverse side effects [95]. Although no epigenetic medications are FDA-approved for pharmacological treatment in SUD [96], there are a few ongoing phase I and II clinical trials for evaluating SUD, suggesting that humans could tolerate these limited side effects of epigenetic therapeutic intervention [95]. In order to make epigenetic pharmacotherapy become a feasible clinical approach for SUD, in-depth investigations need to be completed to ameliorate the specificity and selectivity of epigenetic inhibitors in compulsive drug-seeking behaviors of human addicts.

**Table 2:** Histone modifying enzyme inhibitors in substance use disorders.

Drug	Epigenetic inhibitor	Model	Targeted enzyme	Behavioral effect	Reference
**Cocaine**	TSA	Rats, intravenous injection	HDACs	SA ↓	[119]
		Rats, NAcSh bilateral microinjections	HDACs	SA ↑	[103]
	Garcinol	Rats, systemic injection	HATs	SA reinstatement ↓	[120]
	RGFP-966	Rats, systemic treatment	HDAC3	SA extinction ↑	[47]
		Rats, systemic treatment	HDAC3	CPP extinction ↑	[50]
	Phenylbutyrate	Rats, intravenous injection	HDACs	SA ↓	[119]
	JQ1	Mice, intracranial injection	BETs	CPP acquisition ↓	[53]
		Mice, intra-NAc injection	BETs	CPP reinstatement ↓ SA ↓	[121]
	SKLB-639	Mice, intra-NAc injection	PRMT1	Cocaine-induced behavior ↓	[44]
	GSK-J4	Mice, systemic injection	KDM6B	CPP reinstatement ↓	[122]
	BIX01294	Mice, intra-NAc injection	GLP/G9a	CPP acquisition ↓	[115]
	Sirtinol	Mice, intra-NAc injection	Sirtuins	CPP acquisition ↓	[102]
	NaB	Mice, systemic injection	HDACs	low-dose, CPP extinction ↑ high-dose, CPP extinction ↓	[123]
	RVX-208	Mice, systemic injection	BD2	CPP ↓	[56]
**METH**	JQ1	Mice, systemic injection	BETs	CPP acquisition ↓	[93]
	NaB	Mice, systemic injection	HDACs	Locomotor response ↑	[109]
	VPA	Rats, single intracerebral injection	HDACs	Locomotor response ↓	[124]
	MeBib	Rats, intraperitoneal injection	HDAC6	SA ↓	[125]
**Morphine**	NaB	Rats, systemic injection	HDACs	CPP extinction ↑ CPP reinstatement ↓	[126]
		Mice, systemic injection	HDACs	CPP acquisition ↑	[127]
	TSA	Rats, intracerebral microinjection	HDACs	morphine-induced behavior ↓	[108]
	VPA	Mice, systemic injection	HDACs	locomotor sensitization ↓	[128]
**Heroin**	NaB	Rats, systemic injection	HDACs	Heroin-seeking reinstatement ↑	[89]
	JQ1	Rats, intra-dStri injection	BETs	Heroin-induced behavior ↓	[88]

Abbreviation.

BETs: bromodomain and extra terminal domain.

CPP: conditioned place preference.

dStri: dorsal striatum.

GLP: G9a-like protein.

HATs: histone acetyltransferases.

HDACs: histone deacetylases.

KDM6B: lysine demethylase 6B

NaB: sodium butyrate.

NAc: nucleus accumbens.

NAcSh: nucleus accumbens shell.

PRMT1: protein arginine methyltransferase 1.

SA: self-administration.

TSA: Trichostatin A.

VPA: valproic acid.

Although the understanding of how neuroplastic alterations occur in brain regions is gradually becoming abundant, there is still a tremendous lack of the individual roles of histone modifications in complex brain structures, especially in single neuronal subtypes and non-neuronal cells. Hence, it is paramount to separate and examine these heterogeneous cell populations, and further the understanding of individual cell types in SUD. Till now, the cell type-specific epigenetic manner has been investigated through new gene- and cell-type-specific epigenetic techniques, including gene editing, 3D brain organoids and next-generation sequencing at a single cell level [97]. For instance, single-cell RNA sequencing (scRNA-seq) has been utilized to dissect the cellular diversity of brain regions and study drug-induced differential gene expression in cellular composition and made definite progress [98]. Recently, spatial omics methods have enabled characterization of chromatin accessibility and histone modifications in individual cell [99]. Overall, addressing the interaction with epigenome will substantially advance our understanding of the molecular bases underlying the formation, persistence, withdrawal and relapse of SUD. It might underlie the transition from early stages of recreational drug-taking behaviors to subsequent compulsive use stages when the standards of SUD are met. Further studies are necessary to develop a comprehensive model of how histone modification contributes to the SUD process and generate effective treatment strategies.

## Author contribution statement

All authors listed have significantly contributed to the development and the writing of this article.

## Data availability statement

Data included in article/supp. material/referenced in article.

## Declaration of competing interest

The authors declare there is no potential conflict of interests.
